# Evidence for managing quality and financial health for sustainability

**Published:** 2019-05-13

**Authors:** Umang Mathur, Suneeta Dubey, Deepika Verma

**Affiliations:** 1 Executive Director: Dr. Shroff's Charity Eye Hospital, New Delhi, India; 2 Medical Superintendent: Head of Department -Quality Assurance, Dr. Shroff's Charity Eye Hospital, New Delhi, India; 3 Quality Manager: Dr. Shroff's Charity Eye Hospital, New Delhi, India

**Quality improvement (QI) initiatives should be based on sound evidence to be effective since human resources, efforts and a lot of time is invested into the process.**

**Figure:**
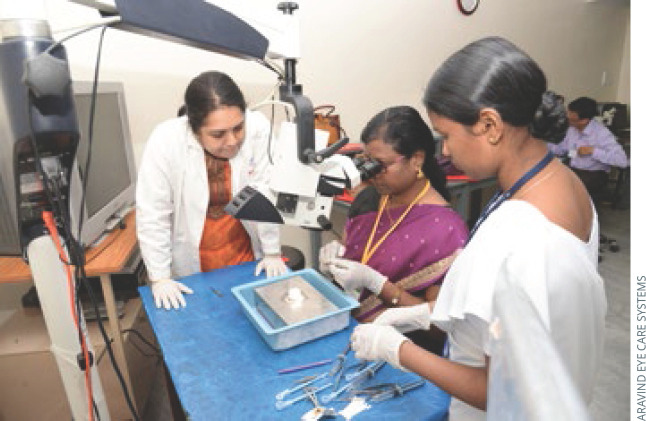
Continuous quality improvement is crucial for patient satisfaction. INDIA

Continuous quality improvement is crucial for patient satisfaction which subsequently leads to financial viability. Quality improvement (QI) initiatives should be based on sound evidence to be effective since human resources, efforts and a lot of time is invested into the process. Also, quality improvement processes affect the financial health of an organisation.

Sackett et al defined evidence-based medicine (EBM) as “the conscientious, explicit, and judicious use of current best evidence in making decisions about the care of individual patients, integrating individual clinical expertise with the best available external clinical evidence from systematic research”.^1^ There are two key aspects of this evidence base:

- Quality improvement initiatives should lead to improvements in patient outcomes that are, ideally, both clinically important and cost-effective.^2^
- Quality improvement initiatives should be based on sound evidence of what works to implement these products or approaches.^3^

**Figure 1a:**
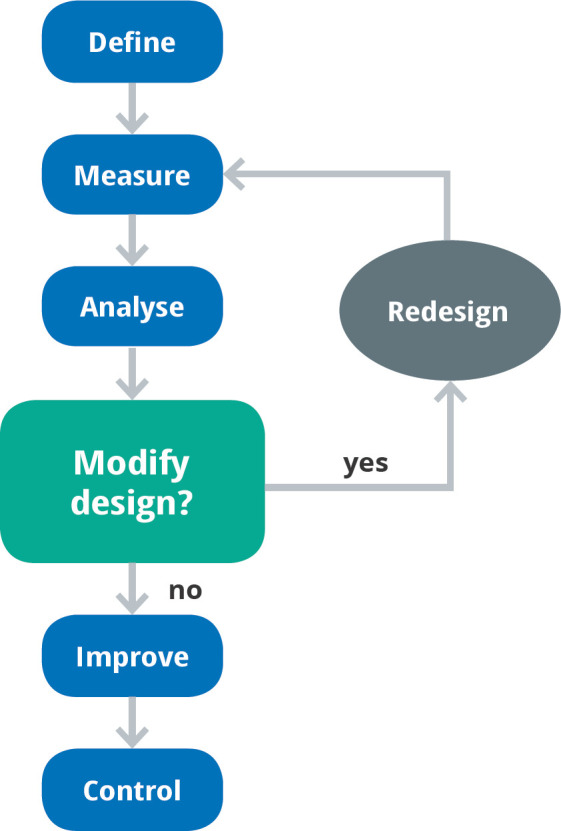
DMAIC strategy

Evidence-based quality improvement (EBQI) requires an evidence-based problem that needs assessment. Also, guidelines governing the process to be improved are essential and there has to be sufficient relevant evidence regarding potential methods for improvement (barriers/facilitators, care models). A team of experts is needed for the propagation of EBQI. EBQI aims to systematically insert evidence, knowledge, and data at all points in development of a QI intervention.

In a reputed National Accreditation Board for Hospitals (NABH) accredited eyecare hospital in North India various EBQI measures are taken in a continuous manner.

A set of quality indicators (quantitative measures that can be used to monitor and evaluate governance, management, clinical, and support functions) are monitored in the hospital regularly (Table 1) which describe the patient or health related outcomes and performance. Patient care is evaluated for consistency through the indicators based on evidence-based standards of care. Every month the top management holds a meeting with the key stakeholders where the data is presented. The improvements, gaps and interventions are discussed in the meetings and crucial decisions are made to make continuous improvements.

**Table 1:** Quality indicators monitored regularly

Patient satisfaction rate (benchmark= 95%) OT starting time (benchmark=90%) Inter-operative time (benchmark= < 10 minutes) Post-operative infection rate (benchmark= <0.08%) Surgical scrubbing rate (benchmark= 100%) Surgical conversion rate (benchmark= 80%) Postponed cases (benchmark= 4%) OPD starting time Medical records completion rate Cataract outcomes Surgical complication rate

**Figure 1b:**
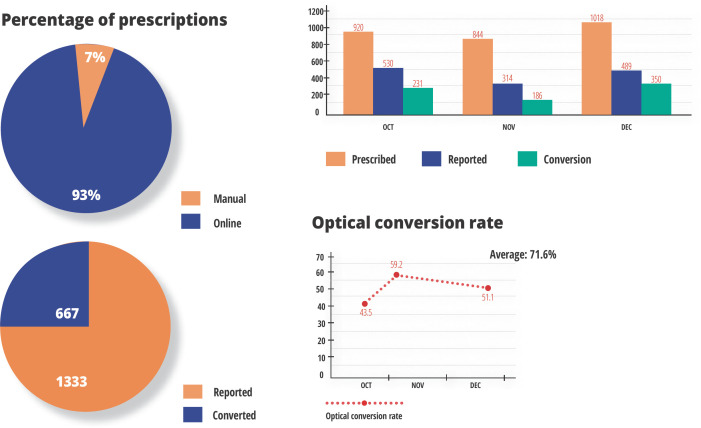
Illustrates the improved outcome after evidence-based quality improvement initiative undertaken in project Chakshjyoti.

The standard operating procedures of all the departments of the hospital are reviewed and updated each year by the technical experts. Latest national and international guidelines are used for this purpose as evidence.

The quality team of the hospital also takes up various lean Six Sigma projects for continuous quality improvement. These initiatives are based on the evidence and effectiveness of DMAIC strategy. DMAIC is a data driven quality strategy used to improve processes. It is an integral part of a Six Sigma initiative, but in general can be implemented as a standalone quality improvement procedure. DMAIC is an acronym for five phases that make up the process (Figure 1a)^4^:

- Define the problem, improvement activity, opportunity for improvement, the project goals, and customer (internal and external) requirements.
- Measure process performance.
- Analyse the process to determine root causes of variation, poor performance (defects).
- Improve process performance by addressing and eliminating the root causes.
- Control the improved process and future process performance.

In the year 2016, continuous quality improvement projects were on:

- Project Chakshjyoti: to improve the quality of optical services and optical conversion rate (Fig. 1b);
- Project to improve surgical conversion rate;
- Project to reduce patient waiting time in out patient department (OPD)

These projects were taken up and successfully accomplished with evidence-based DMAIC strategy leading to improved quality of services, patient care and sustained the financial health of the hospital.

The hospital also uses EBQI tools like “audit and feedback” to improve its quality of services. A prescription error audit was also done at the hospital in the last financial year taking MCI (Medical Council of India) guidelines as evidence. The results were communicated to the clinicians and post intervention a re-audit was done which showed significant improvement in the system.

Operating theatre starting time is also monitored at all the secondary centres of the hospital. At one of the secondary centres it was showing higher non-compliance consistently. NABH guidelines make it mandatory to have hospital committees to ensure patient safety and prevention from hospital-acquired infections. Evidence suggests that these committees are essential and play an important role in patient care.

Hence, such examples illustrate that quality and evidence-based practice are mutually linked. To make quality improvement interventions effective, evidence-based methods are essential. This ultimately leads to improved patient care along with patient satisfaction and sustained financial health of the organisation.

## References

[1] Sackett DL Rosenberg WM Gray JA Haynes RB Richardson WS. Evidence-based medicine: what it is and what it isn't. Clinical Orthopedics and Related Research 2007; 455:3—5. 17340682

[2] Cooksey D. A Review of UK Health Research Funding. Stationary Office: London, 2006.

[3] U.S. Department of Veteran Affairs. Evidence Based Quality Improvement (EBQI): Can this approach be helpful in improving healthcare for women Veterans? https://www.hsrd.research.va.gov/for_researchers/cyber_seminars/archives/video_archive.cfm?SessionID=425 (accessed 5 February 2018)

[4] Borror C.M. The Certified Quality Engineer Handbook. ASQ Quality Press; 2009.

